# Supplementary material for: An improved primer set and amplification protocol with increased specificity and sensitivity targeting the Symbiodinium ITS2 region
Source: PeerJ. 2018 May 23;6:e4816. doi: 10.7717/peerj.4816 (PMC5970565; doi:10.7717/peerj.4816)
Supplement: Supplemental Information 1 [file peerj-06-4816-s001.docx]

**Supplementary Information**

**An improved primer set and amplification protocol with increased specificity and sensitivity targeting the *Symbiodinium* ITS2 region**

Benjamin CC Hume^1^†, Maren Ziegler^1^, Julie Poulain^2,3,4^, Xavier Pochon^5,6^, Sarah Romac^7^, Emilie Boissin^8^, Colomban de Vargas^7^, Serge Planes^8^, Patrick Wincker^2,3,4^, Christian R Voolstra^1^†

^1^Red Sea Research Center, Division of Biological Sciences and Engineering (BESE), King Abdullah University of Science and Technology (KAUST), Thuwal, Saudi Arabia

^2^CEA - Institut de Biologie François Jacob, Genoscope, 2 rue Gaston Crémieux, 91057 Evry, France.

^3^ CNRS, UMR 8030, CP5706, Evry, France.

^4^ Université d'Evry, UMR 8030, CP5706, Evry, France.

^5^ Coastal and Freshwater Group, Cawthron Institute, Private Bag 2, Nelson 7042, New Zealand.

^6^ Institute of Marine Science, University of Auckland, Private Bag 349, Warkworth 0941, New Zealand.

^7^ CNRS, UMR 7144, EPEP & Sorbonne Universités, UPMC Université Paris 06; Station Biologique de Roscoff, 29680 Roscoff, France.

^8^ PSL Research University: EPHE-UPVD-CNRS, USR 3278 CRIOBE, Université de Perpignan, 52 Avenue Paul Alduy, 66860 Perpignan Cedex, France

†Corresponding authors:

Christian R Voolstra

Bldg 2, Rm 2226

Red Sea Research Center, KAUST

Phone: +966-12-808 2377

Email: [christian.voolstra@kaust.edu.sa](mailto:christian.voolstra@kaust.edu.sa)

Benjamin C C Hume

Bldg 2, Rm 2227 WS01

Red Sea Research Center, KAUST

Phone: +966-544-701-291

Email: [benjamin.hume@kaust.edu.sa](mailto:benjamin.hume@kaust.edu.sa)

Running title: Assessment of *Symbiodinium* ITS2 primer pairs

## Supplementary Tables

## Supplementary Table 1 Sample details and PCR conditions. Samples analysed for primer pair comparisons and optimisations are denoted.

| Sample Name | | Environment^1^ | Primer pair | Cycle No. | | Annealing temp ^o^C | Post-QC seqs | *Symbiodinium* seqs | PCR UID |
| --- | --- | --- | --- | --- | --- | --- | --- | --- | --- |
|  |  |  |  |  |  |  |  |  |  |
| CO-0000150 | | CO | SYM_VAR | 35 | | 56 | 37700 | 37700 | BUR_AAEB |
| CO-0000151 | | CO | SYM_VAR | 35 | | 56 | 36399 | 36399 | BUR_AAEC |
| CO-0000208 | | CO | SYM_VAR | 35 | | 56 | 30683 | 30683 | BUR_AAEF |
| CO-0000209 | | CO | SYM_VAR | 35 | | 56 | 37372 | 37372 | BUR_AAEG |
| CO-0000302 | | CO | SYM_VAR | 35 | | 56 | 22549 | 22549 | BUR_AAED |
| CO-0000303 | | CO | SYM_VAR | 35 | | 56 | 23214 | 23214 | BUR_AAEE |
| AW-0000035 | | SRF-CO | SYM_VAR | 35 | | 56 | 23396 | 9983 | BUM_AADC |
| IW-0000015 | | CSW | SYM_VAR | 35 | | 56 | 25124 | 25113 | BUM_AADD |
| IW-0000021 | | CSW | SYM_VAR | 35 | | 56 | 35726 | 34268 | BUM_AADE |
| OA-0000044 | | SRF-OO | SYM_VAR | 35 | | 56 | 33039 | 0 | BUM_AACX |
| OA-0000058 | | SRF-OO | SYM_VAR | 35 | | 56 | 22873 | 0 | BUM_AACY |
| OA-0000106 | | SRF-OO | SYM_VAR | 35 | | 56 | 30385 | 0 | BUM_AACZ |
| OA-0000118 | | SRF-OO | SYM_VAR | 35 | | 56 | 28149 | 0 | BUM_AACW |
| OA-0000218 | | SRF-OO | SYM_VAR | 35 | | 56 | 24378 | 5987 | BUM_AADA |
| OA-0000280 | | SRF-OO | SYM_VAR | 35 | | 56 | 22421 | 153 | BUM_AADB |
| N000001272 | | SRF-OO | SYM_VAR | 35 | | 56 | 20459 | 0 | BZM_AABT |
| N000001265 | | SRF-OO | SYM_VAR | 35 | | 56 | 33837 | 194 | BZM_AABQ |
| N000001578 | | SRF-OO | SYM_VAR | 35 | | 56 | 22566 | 374 | BZM_AABF |
| N000001943 | | SRF-OO | SYM_VAR | 35 | | 56 | 20183 | 9263 | BZM_AABI |
| N000001994 | | SRF-OO | SYM_VAR | 35 | | 56 | 10691 | 1948 | BZM_AABK |
| N000002039 | | SRF-OO | SYM_VAR | 35 | | 56 | 23385 | 1415 | BZM_AABM |
| N000002021 | | SRF-OO | SYM_VAR | 35 | | 56 | 28089 | 11552 | BZM_AABO |
| N000002137 | | SRF-OO | SYM_VAR | 35 | | 56 | 26240 | 119 | BZM_AABV |
| N000002699 | | SRF-OO | SYM_VAR | 35 | | 56 | 23077 | 67 | BZM_AABZ |
| N000002745 | | SRF-OO | SYM_VAR | 35 | | 56 | 24961 | 39 | BZM_AABX |
| N000000073 | | SRF-OO | SYM_VAR | 35 | | 56 | 21909 | 228 | BZM_AABB |
| N000000278 | | SRF-OO | SYM_VAR | 35 | | 56 | 21738 | 0 | BZM_AABD |
|  | |  |  |  | |  |  |  |  |
| CO-0000150 | | CO | ITSintfor | 35 | | 56 | 35744 | 25430 | BUR_AADV |
| CO-0000151 | | CO | ITSintfor | 35 | | 56 | 32882 | 26565 | BUR_AADW |
| CO-0000208 | | CO | ITSintfor | 35 | | 56 | 30005 | 30005 | BUR_AADZ |
| CO-0000209 | | CO | ITSintfor | 35 | | 56 | 27343 | 26740 | BUR_AAEA |
| CO-0000302 | | CO | ITSintfor | 35 | | 56 | 47260 | 37622 | BUR_AADX |
| CO-0000303 | | CO | ITSintfor | 35 | | 56 | 44946 | 36594 | BUR_AADY |
| AW-0000035 | | SRF-CO | ITSintfor | 35 | | 56 | 18759 | 161 | BUM_AACT |
| IW-0000015 | | CSW | ITSintfor | 35 | | 56 | 21678 | 14183 | BUM_AACU |
| IW-0000021 | | CSW | ITSintfor | 35 | | 56 | 16917 | 5685 | BUM_AACV |
| OA-0000044 | | SRF-OO | ITSintfor | 35 | | 56 | 23885 | 0 | BUM_AACO |
| OA-0000058 | | SRF-OO | ITSintfor | 35 | | 56 | 26046 | 0 | BUM_AACP |
| OA-0000106 | | SRF-OO | ITSintfor | 35 | | 56 | 26785 | 0 | BUM_AACQ |
| OA-0000118 | | SRF-OO | ITSintfor | 35 | | 56 | 19409 | 0 | BUM_AACN |
| OA-0000218 | | SRF-OO | ITSintfor | 35 | | 56 | 17923 | 291 | BUM_AACR |
| OA-0000280 | | SRF-OO | ITSintfor | 35 | | 56 | 17058 | 0 | BUM_AACS |
| N000001272 | | SRF-OO | ITSintfor | 35 | | 56 | 20337 | 0 | BZM_AAAT |
| N000001265 | | SRF-OO | ITSintfor | 35 | | 56 | 24028 | 0 | BZM_AAAQ |
| N000001578 | | SRF-OO | ITSintfor | 35 | | 56 | 22415 | 18 | BZM_AAAF |
| N000001943 | | SRF-OO | ITSintfor | 35 | | 56 | 25793 | 17 | BZM_AAAI |
| N000001994 | | SRF-OO | ITSintfor | 35 | | 56 | 19850 | 7 | BZM_AAAK |
| N000002039 | | SRF-OO | ITSintfor | 35 | | 56 | 19262 | 10 | BZM_AAAM |
| N000002021 | | SRF-OO | ITSintfor | 35 | | 56 | 21170 | 21 | BZM_AAAO |
| N000002137 | | SRF-OO | ITSintfor | 35 | | 56 | 15653 | 0 | BZM_AAAV |
| N000002699 | | SRF-OO | ITSintfor | 35 | | 56 | 15905 | 0 | BZM_AAAZ |
| N000002745 | | SRF-OO | ITSintfor | 35 | | 56 | 17682 | 0 | BZM_AAAX |
| N000000073 | | SRF-OO | ITSintfor | 35 | | 56 | 15014 | 7 | BZM_AAAB |
| N000000278 | | SRF-OO | ITSintfor | 35 | | 56 | 14537 | 0 | BZM_AAAD |
|  | |  |  |  | |  |  |  |  |
| CO-0000150 | | CO | ITS-DINO | 35 | | 56 | 34769 | 21190 | BUR_AAEN |
| CO-0000151 | | CO | ITS-DINO | 35 | | 56 | 22345 | 16076 | BUR_AAEO |
| CO-0000208 | | CO | ITS-DINO | 35 | | 56 | 22504 | 21646 | BUR_AAER |
| CO-0000209 | | CO | ITS-DINO | 35 | | 56 | 20937 | 15865 | BUR_AAES |
| CO-0000302 | | CO | ITS-DINO | 35 | | 56 | 33311 | 22995 | BUR_AAEP |
| CO-0000303 | | CO | ITS-DINO | 35 | | 56 | 34745 | 26870 | BUR_AAEQ |
| AW-0000035 | | SRF-CO | ITS-DINO | 35 | | 56 | 14815 | 83 | BUM_AADU |
| IW-0000015 | | CSW | ITS-DINO | 35 | | 56 | 24269 | 13535 | BUM_AADV |
| IW-0000021 | | CSW | ITS-DINO | 35 | | 56 | 17993 | 3899 | BUM_AADW |
| OA-0000044 | | SRF-OO | ITS-DINO | 35 | | 56 | 14810 | 0 | BUM_AADP |
| OA-0000058 | | SRF-OO | ITS-DINO | 35 | | 56 | 19337 | 0 | BUM_AADQ |
| OA-0000106 | | SRF-OO | ITS-DINO | 35 | | 56 | 22648 | 0 | BUM_AADR |
| OA-0000118 | | SRF-OO | ITS-DINO | 35 | | 56 | 17452 | 0 | BUM_AADO |
| OA-0000218 | | SRF-OO | ITS-DINO | 35 | | 56 | 14819 | 149 | BUM_AADS |
| OA-0000280 | | SRF-OO | ITS-DINO | 35 | | 56 | 23588 | 3 | BUM_AADT |
| N000001272 | | SRF-OO | ITS-DINO | 35 | | 56 | 13524 | 0 | BZM_AADT |
| N000001265 | | SRF-OO | ITS-DINO | 35 | | 56 | 15242 | 0 | BZM_AADQ |
| N000001578 | | SRF-OO | ITS-DINO | 35 | | 56 | 12282 | 3 | BZM_AADF |
| N000001943 | | SRF-OO | ITS-DINO | 35 | | 56 | 18475 | 303 | BZM_AADI |
| N000001994 | | SRF-OO | ITS-DINO | 35 | | 56 | 18727 | 23 | BZM_AADK |
| N000002039 | | SRF-OO | ITS-DINO | 35 | | 56 | 27063 | 51 | BZM_AADM |
| N000002021 | | SRF-OO | ITS-DINO | 35 | | 56 | 19279 | 227 | BZM_AADO |
| N000002137 | | SRF-OO | ITS-DINO | 35 | | 56 | 12374 | 0 | BZM_AADV |
| N000002699 | | SRF-OO | ITS-DINO | 35 | | 56 | 14831 | 0 | BZM_AADZ |
| N000002745 | | SRF-OO | ITS-DINO | 35 | | 56 | 14500 | 0 | BZM_AADX |
| N000000073 | | SRF-OO | ITS-DINO | 35 | | 56 | 11961 | 23 | BZM_AADB |
| N000000278 | | SRF-OO | ITS-DINO | 35 | | 56 | 11045 | 0 | BZM_AADD |
|  | |  |  |  | |  |  |  |  |
| CO-0000150 | | CO | SYM_VAR | | 25 | 56 | 99626 | 99626 | BUR_AADG |
| CO-0000151 | | CO | SYM_VAR | | 25 | 56 | 102149 | 102149 | BUR_AADH |
| CO-0000208 | | CO | SYM_VAR | | 25 | 56 | 92663 | 92663 | BUR_AADK |
| CO-0000209 | | CO | SYM_VAR | | 25 | 56 | 106830 | 106240 | BUR_AADL |
| CO-0000302 | | CO | SYM_VAR | | 25 | 56 | 99784 | 99784 | BUR_AADI |
| CO-0000303 | | CO | SYM_VAR | | 25 | 56 | 128780 | 127641 | BUR_AADJ |
| AW-0000035 | | SRF-CO | SYM_VAR | | 25 | 56 | 91733 | 25653 | BUM_AACH |
| IW-0000015 | | CSW | SYM_VAR | | 25 | 56 | 65193 | 61975 | BUM_AACI |
| IW-0000021 | | CSW | SYM_VAR | | 25 | 56 | 123100 | 110544 | BUM_AACJ |
|  | |  |  | |  |  |  |  |  |
| AW-0000035 | | SRF-CO | SYM_VAR | | 25 | 56 | 125313 | 31222 | BUM_AADZ |
| IW-0000015 | | CSW | SYM_VAR | | 25 | 56 | 273564 | 265445 | BUM_AAEA |
| OA-0000044 | | SRF-OO | SYM_VAR | | 25 | 56 | 284897 | 0 | BUM_AADX |
| OA-0000058 | | SRF-OO | SYM_VAR | | 25 | 56 | 253396 | 0 | BUM_AADY |
|  | |  |  | |  |  |  |  |  |
| AW-0000035 | | SRF-CO | SYM_VAR | | 25 | 59 | 251041 | 117075 | BUM_AAED |
| IW-0000015 | | CSW | SYM_VAR | | 25 | 59 | 228530 | 224950 | BUM_AAEE |
| OA-0000044 | | SRF-OO | SYM_VAR | | 25 | 59 | 151854 | 0 | BUM_AAEB |
| OA-0000058 | | SRF-OO | SYM_VAR | | 25 | 59 | 364353 | 0 | BUM_AAEC |
|  | |  |  | |  |  |  |  |  |
| CO-0000150 | | CO | Polymerase^2^ | | 30 | 56 | 68685 | 68383 | BUR_AAEZ |
| CO-0000151 | | CO | Polymerase | | 30 | 56 | 79193 | 79193 | BUR_AAFA |
| CO-0000208 | | CO | Polymerase | | 30 | 56 | 76413 | 76413 | BUR_AAFD |
| CO-0000209 | | CO | Polymerase | | 30 | 56 | 54263 | 54097 | BUR_AAFE |
| CO-0000302 | | CO | Polymerase | | 30 | 56 | 69640 | 69640 | BUR_AAFB |
| CO-0000303 | | CO | Polymerase | | 30 | 56 | 88228 | 88228 | BUR_AAFC |
| AW-0000035 | | SRF-CO | Polymerase | | 30 | 56 | 42646 | 7434 | BUM_AAEH |
| IW-0000015 | | CSW | Polymerase | | 30 | 56 | 68150 | 60596 | BUM_AAEI |
| IW-0000021 | | CSW | Polymerase | | 30 | 56 | 60893 | 42214 | BUM_AAEJ |
| OA-0000044 | | SRF-OO | Polymerase | | 30 | 56 | 43000 | 0 | BUM_AAEF |
| OA-0000058 | | SRF-OO | Polymerase | | 30 | 56 | 89563 | 0 | BUM_AAEG |
|  | |  |  | |  |  |  |  |  |

^1^ – Environment abbreviations: CO, coral; CSW, coral surrounding water; SRF, surface water; OA, open ocean.

^2^ – Samples with the primer pair listed as Polymerase were amplified with the SYM_VAR primers but using the Advantage 2 rather than the Phusion polymerase system.

**Supplementary Figures**

**Supplementary Figure 1. Mean sequences returned per sample and proportion retained during the quality control pipeline (QC) in relation to primer pair used.** The number of sequences retained at three stages of the QC pipeline are given: ‘raw_contigs’ (number of sequences returned from mothur’s *make.contigs* command), ‘post-qc’ (the number of sequences remaining after all quality control and taxonomic identification), and ‘*Symbiodinium’* (the number of sequences identified as *Symbiodinium*). Samples are grouped according to their environment and amplification primers. Abbreviations as follows: CO – coral tissue, CSW – coral surrounding water, SRF-CO – coral reef surface water, SRF-OO – open ocean surface water.

**Supplementary Figure 2**. **Comparison and taxonomic composition of sequences returned using three *Symbiodinium* ITS2 primer pairs before removal of sequences JN406302 and JN406301.** Each plot represents one sample and contains three pairs of stacked columns, one for each primer pair in the order of: SYM_VAR, ITSintfor, ITS-DINO (from left to right). For each pair of stacked columns, the left coloured bar denotes the taxonomic breakdown of all returned sequences into 7 categories, and the right greyscale bar denote the sub-genus (cladal) distribution for the subset of sequences classified as *Symbiodinium*. Plots are annotated with the number of sequences returned after QC and the proportion of those sequences that were annotated as Symbiodinium underneath the coloured and grayscale bars, respectively. For each environment type (indicated to the left of the plot groupings) a summary plot is given at the right side, showing the average taxonomic breakdowns, total post-QC sequences returned, and average proportion of *Symbiodinium* sequences.

**Supplementary data**

**Supplementary Data 1 – The fasta sequences used to generate the *Symbiodinium* cladal database.**

>cladeE

AACCAATAGCACCCTGAACTCGCATTGCACTCTTGGGACACGCCTGAGAGTATGTCTGCTTCAGTGCTTTTCATATCTTCGCAGTGCGGGCTTCCTGGAGAAGCCTTGAGCCTCTTTGTGTGCTGCTGCATCAGAATTTGCAGCGGCGCGCTGAACACAAACCGGGAGGTAAGCTGGACTGATTTGTCGCGCATCACTGGGTACGTGTGTCCGTTTTGGCCCAATCATGCCAGCCTGCCAAGCAATTGGTGCTCAATTACCAATCT

>cladeG

AACCAATGGCCTCCTGAACGCGCATTGCACTCTTGGGCTTCCCTGAGAGTATGTTTGCTTCAGTGCTTCTTTTGCTCAACCATTGCAAGGTTTGGCAGTGCAATGCCTCCCTGTGCCTCGGCGTGTTGTTGGCGTGTCTGCCAATGACGTGCGACCAGCGTGGCCTATGTGCAAGCATGCACGTGCTTTGTTGTTTCACTGCAGACATTCTCCGGAATATGCGTGGGCGACGTGGCTGATGCTTGCGGACGCGCTAGTGTGCTGCTTGCACTTCTTCCATAGCATGAAGTCAAACAGGCA

>cladeA

AACCAATGGCCTCTTGAACGTGCATTGCGCTCTTGGGATATGCCTGAGAGCATGTCTGCTTCAGTGCTTCTACTTTCATTTTCTGCTGCTCTTGTTATCAGGAGCAGTGTTGCTGCATGCTTCTGCAAGTGGCACTGGCATGCTAAATATCAAGTTTTGCTTGCTGTTGTGACTGATCAACATCTCATGTCGTTTCAGTTGGCGAAACAAAAGCTCATGTGTGTTCTTAACACTTCCTAGCATGAAGTCAGACAAGTGAACCCCAGACAAGTGA

>cladeI

AACCAATGGCCTCCTGAACGCTCATTGCACCCTTGGGATTTCCTGAGGGCATGTCTGCTTCAGTGCTTAGCTTTTACACCTTCGTGCGGGCGCGATGTTTTCGTGTCCTGCACTCCTGCAAGCCATCGCTCAGATTTGCTTCTGATGGCTTGTTGAATGATTGGCTGTTTTGCAAGCTCAAGCGCTTTGTGATTCATAGCAAACCTATGGGATTCGCTTGGGTCGCGCTGCTGATGCCTACAGCCTTCAGCATGTGAAACCGCATGCATCTTAGCATGAAGTCAGACAAGAGA

>cladeD

AACCAATGGCCCCGTGAACGCGCATTGCACTCTTGGGACTTCCTGAGAGTATGTTTGCTTCAGTGCTTATTTTACCTCCTTGCAAGGTTCTGTCGCAACCTTGTGCCCTGGCCAGCCACGGGTTAACTTGCCCATGGCTTGCTGAGTAGTGATCTTTTAGAGCAAGCTCTGGCACGCTGTTGTTTGAGGCAGCCTATATTGAGGCTATTTCAAATGACGTTGCTACAAGCTTGATGTGTCCTTCTGCGCCGTTGCGCATCCCATAGCATGAAGTCAAACAAGAGA

>cladeB

AACCGATGGCCTCCTGAACGCGCATTGCGCTCTCGGGATTTCCTGAGAGCAGGTCTGCTTCAGTGCTTAGCATTATCTACCTGTGCTTGCAAGCAGCATGTATGTCTGCATTGCTGCTTCGCTTTCCAACAAGTCATCGATCGCTTTTGTGTTCGTAAATGGCTTGTTTGCTGCCTGGCCCATGCGCCAAGCTTGAGCGTACTGTTGTTCCAAGCTTTGCTTGCATCGTGCAGCTCAAGCGCGCAGCTGTCGGGATGCTGATGCATGCCCTTAGCATGAAGTCAGACAAGAG

>cladeF

AATCAATGGCCTCCTGAACGTACGTTGCACTCTTGGGGTTTCCTGAGAGTATGTCTGCTTCAGTGCTTAGCATGCATAACCCTGCGAGCAGTTTTGTTTGCTTTGCGCTTTTATGAGCCATTGGTTTCCAGCCAATGGCTTGTTAAATAGTTTTTTGCAAATGAAAGCTCTGCGCGCTGTTGTTCAAGCAAGTGCCTTTCAGGTTTCTAGGCTTGAGTGACGCTGCTCATGCTTGCAACTGCCAGGCTGCCAGTGCACGCCTCTAGCATGAAGTCAGGCAAGTGA

>cladeC

AACCAATGGCCTCCTGAACGTGCGTTGCACTCTTGGGATTTCCTGAGAGTATGTCTGCTTCAGTGCTTAACTTGCCCCAACTTTGCAAGCAGGATGTGTTTCTGCCTTGCGTTCTTATGAGCTATTGCCCTCTGAGCCAATGGCTTGTTAATTGCTTGGTTCTTGCAAAATGCTTTGCGCGCTGTTATTCAAGTTTCTACCTTCGTGGTTTTACTTGAGTGACGTTGCTCATGCTTGCAACCGCTGGGATGCAGGTGCATGCCTCTAGCATGAAGTCAGACAAGTGA

>cladeH

GCTTCAGTGCTTAGCTTACCCAACTTTGCAAGCAGGCTTAAAGTCTGCGTCGCGTTCCTATGAGCTATTGTGCTTCCTTTGCCAATGGCTTGTTGAGTGGTAGGTTCCTGCAAAATGCTTTGCGCGCTGTTATTCAAGTTTCGCCTGCACGGCTTTGCTTGAGTGACGCTGCTCATGCATGCAACCGCTGGGGTGCGCTCTGCGCATGCCTCTAGCATGAAGTCAGACAAGCGAACCCG
